# The heart healthy lenoir project-an intervention to reduce disparities in hypertension control: study protocol

**DOI:** 10.1186/1472-6963-13-441

**Published:** 2013-10-25

**Authors:** Jacquie R Halladay, Katrina E Donahue, Alan L Hinderliter, Doyle M Cummings, Crystal W Cene, Cassie L Miller, Beverly A Garcia, Jim Tillman, Darren DeWalt

**Affiliations:** 1 Department of Family Medicine, University of North Carolina at Chapel Hill, 590 Manning Drive, Chapel Hill, NC 27599, USA; 2 Center for Health Promotion and Disease Prevention, UNC Chapel Hill, Chapel Hill, NC 27599, USA; 3 Department of Medicine, UNC Chapel Hill, Chapel Hill, NC 27599, USA; 4 Department of Family Medicine, East Carolina University, Greenville, NC 27858, USA; 5 Cecil G. Sheps Center for Health Services Research, Chapel Hill, NC 27599, USA; 6 Community Care Plan of Eastern North Carolina, Greenville, NC 27858, USA

**Keywords:** Hypertension, Methods, Quality improvement, Disparities, Community based participatory research

## Abstract

**Background:**

Racial disparities in blood pressure control are well established; however the impact of low health literacy (LHL) on blood pressure has garnered less attention. Office based interventions that are created with iterative patient, practice and community stakeholder input and are rolled out incrementally, may help address these disparities in hypertension control. This paper describes our study protocol.

**Methods/design:**

Using a community based participatory research (CBPR) approach, we designed and implemented a cohort study that includes both a practice level and patient level intervention to enhance the care and support of patients with hypertension in primary care practices in a rural region of eastern North Carolina. The study is divided into a formative phase and an ongoing 2.5 year implementation phase. Our main care enhancement activities include the integration of a community health coach, using home blood pressure monitoring in clinical decision making, standardizing care delivery processes, and working to improve medication adherence. Main outcomes include overall blood pressure change, the differential change in blood pressure by race (African American vs. White) and health literacy level (low vs. higher health literacy).

**Discussion:**

Using a community based participatory approach in primary care practice settings has helped to engage patients and practice staff and providers in the research effort and in making practice changes to support hypertension care. Practices have engaged at varying levels, but progress has been made in implementing and iteratively improving upon the interventions to date.

**Trial registration:**

ClinicalTrials.gov NCT01425515.

## Background

Hypertension affects more than 67 million persons in the US [1] and increases the risk of all-cause mortality, mortality due to heart disease, stroke, chronic kidney disease, and heart failure, and morbidity associated with non-fatal cardiovascular disease [2]. Although treating and controlling hypertension is the single most effective clinical service for reducing mortality [3], blood pressure is currently not controlled in more than half of American adults with this condition [1].

Compared with Whites, African Americans have a higher adjusted prevalence of hypertension (40.1% vs. 27.4%) [4], and are more likely to be aware of and receive treatment for their disease [4]. But, African Americans continue to have lower rates of hypertension control compared with Whites (33.4% vs. 36.8%) [4]. African Americans acquire the disease at younger ages [5] and have higher rates of severe hypertension than Whites [5,6]. These factors contribute to an 80% higher stroke mortality rate, 50% higher heart disease mortality rate, and a 320% greater rate of hypertension-related end stage renal disease than in the general population [7,8].

Racial disparities in blood pressure control are well established. However, the impact of low health literacy (LHL) on blood pressure has garnered less attention [9,10]. LHL makes it more difficult to understand and use health information and may be an obstacle to good BP control. Over 90 million adults lack the literacy skills needed to effectively function in the health care environment [11]. LHL is one of a number of key barriers to following medical and lifestyle hypertension management regimens [12,13] including understanding routine prescription information [14].

To reduce hypertension disparities in hypertension control by race and literacy, we used a community based participatory research (CBPR) approach to design and test an ongoing practice and patient level intervention in a rural region of eastern North Carolina that lies within the “stroke belt”. Our overarching goal is to narrow the gap in blood pressure control between African Americans and Whites and among those with lower and higher health literacy by involving six primary care practices and community partners in a collaborative effort to lower blood pressure and reduce disparities in blood pressure control.

Our main outcomes are overall blood pressure change and the differential change in blood pressure by race (African American vs. White) and health literacy level (low vs. higher health literacy) among people with hypertension. The objective of this paper is to describe our study methods.

## Methods/design

### Study overview

**Figure 1:**
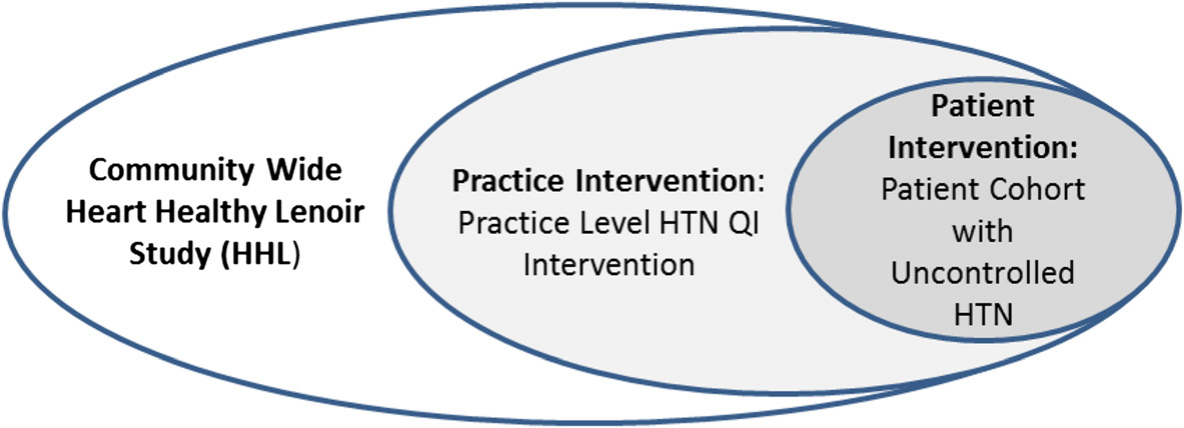
Heart Healthy Lenoir Study Components.

The study is part of an ongoing Heart Healthy Lenoir (HHL) study which is a five-year cardiovascular risk (CVD) reduction project in a geographically defined area in rural Southeastern US. The HHL study started in June 2010 and will run for 5 years. As depicted in Figure 1, the overarching HHL study is a **community level** project that is designed to improve lifestyle, environmental factors, and hypertension control in eastern NC. Six primary care practices within the region are involved in the **practice level** hypertension care quality improvement (QI) intervention study (the Practice Intervention). Within the practices there is an embedded **patient level** cohort study of patients with uncontrolled hypertension (the Patient Intervention). This paper only describes the Practice and Patient Intervention studies which were designed and implemented simultaneously.

**Figure 2:**
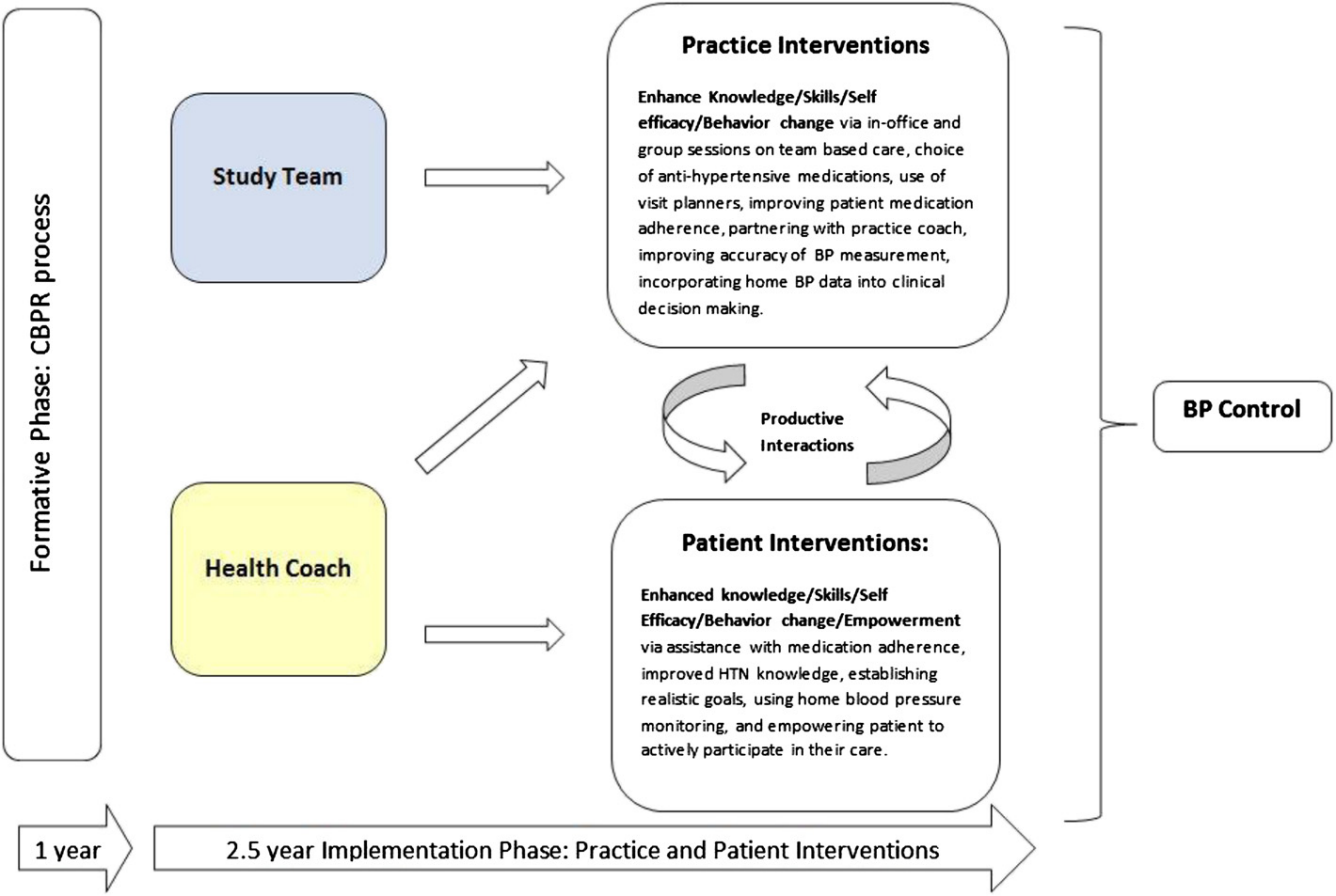
Educational and Behavioral Interventions targeting psychosocial and systems factors to empower practice staff, providers and patients with skills and resources to enhance Blood Pressure (BP) control.

The study is divided into a formative phase that ended in July 2011 and an ongoing 2.5 year implementation phase (see Figure 2). During the formative phase, we interviewed patients, providers, and office staff in order to gain insights into the existing resources and challenges that affect hypertension control in their region. This information then guided the design of the various Practice and Patient Intervention components that are currently being implemented. During the implementation phase, we engage practices on site in their improvement efforts and via formal educational opportunities that we deliver at regional quarterly dinner meetings.

Our Data Safety and Monitoring Board meets every 6 months throughout the project to ensure the safety of the intervention components and provide advice on study related issues. The Biomedical Institutional Review Board at the University of North Carolina reviewed and approved this project.

### Participant recruitment

#### Identification of practices for the practice intervention

To identify practices to invite into the study, we generated lists from the local yellow pages, obtained recommendations from employees at the regional hospital, and received direct recommendations from a provider in one of the primary care practices who has participated in projects with members of our research team in the past.

We initially contacted 8 practices, and 5 agreed to learn more about the study via a face-to-face visit with the study investigators. Four ultimately agreed to participate. To replace the one practice that chose to not participate, we added another practice from this region that we were not aware of at the onset of our recruitment efforts. Thus, 5 primary care practices participated during the formative phase (June 2010-July 2011) and a 6^th^ practice, that was established in 2012, joined during the implementation phase.

Practices were offered $1,000 annually for 5 years for general study participation, $30 per subject enrolled into the Patient Intervention cohort study in order to partially compensate them for their time, and $10 per study-related venipuncture.

### Identifying patients for the patient intervention: recruitment into the study cohort

We worked with practices in the formative phase to find the most feasible way to identify patients with uncontrolled hypertension to participate in the cohort study. Due to differences in a practices’ ability to generate lists of patients with uncontrolled hypertension from their electronic health records (EHR’s), we helped practices create unique mechanisms to identify potential subjects for the study. For practices that could not generate such lists from their EHR’s, practice staff members documented the blood pressure of each adult patient on their daily patient schedules and faxed these lists to our study personnel weekly. In order to create a cohort of patients with uncontrolled hypertension, patients with a systolic BP of 150 mmHg or greater were invited to participate via a mailed letter that was signed by their primary care practice leaders. Approximately one week later, each of these potential participants was called by our study personnel and received an informational “robo call”. The robo call messages were audiotaped using the voice of a practice staff member.

We offered patients a $40 gift card for completing the enrollment visit and provide $25 gift cards for each of 4 follow up visits with the regionally based study team. The subjects recruited into the Patient Intervention received a home blood pressure monitor at their enrollment visit and are provided with a health coach to assist them with their self-management efforts via counseling provided over the phone.

Overall inclusion and exclusion criteria for the Patient Intervention study are listed in Table 1. Patients who were ineligible based upon the results of their laboratory values received a call from one of the study investigators to explain why they could not participate. If deemed ineligible, the subjects kept their blood pressure cuff and initial incentive payment.

**Table 1:** HHL inclusion and exclusion criteria

**Inclusion criteria:**	**Exclusion criteria:**
18 years or older	Non-English speaking
Able and willing to give informed consent	Current treatment for psychosis
A current patient of 1 of the 6 participating practices	Advanced dementia
	Current substance abuse
Diagnosis of hypertension by primary care physician or have 3 documented blood pressures above 150/90 mmHg	Lack of phone access
	History of malignancy, other than non-melanoma
	Skin cancer, that has not been in remission or cured surgically for >5 years
Systolic blood pressure ≥ 150mmHg at their most recent clinic visit	Estimated creatinine clearance ≤ 30 ml/min
Physician approval to participate in the study	Pregnant

### Patient intervention: initial enrollment and follow-up data collection visits

#### Enrollment visit (completed)

Cohort study subjects that remained eligible after a prescreening call met with a research assistant for an initial 1.5-hour enrollment visit. During this visit subjects completed the baseline questionnaire, provided informed consent, had laboratory tests and biometrics measurements obtained and were assessed for health literacy using the Short Test of Functional Health Literacy in Adults (s-TOFHLA). Subjects were provided with either an Omron Model BP 785 electronic home blood pressure monitor (HBPM) with one size cuff or the OMRON BP-652 wrist cuff if their upper arm circumference was greater than 17 inches.

#### Follow-up visits/description of study measurements (ongoing)

The instruments used for all data collection are provided in Table 2. Follow up visits occur at 6,12, 18 or 24 months post enrolment. Biometric measures include height, weight, heart rate, and blood pressure. Our research assistants use the Omron HEM-907 automated blood pressure monitor (Omron Healthcare, Inc., Vernon Hills, IL) and record the average of 3 sequential measurements obtained at 60-second intervals. All blood pressure measurements are taken in the seated position and after a 5 minute rest [9,11]. Weight is measured using a Seca model 770 electronic personal scale (SECA Corporation, Hanover, MD) that has a maximum capacity of 200 kg (440 lb) and accuracy within 50 grams for weights less than 150 kg and within 100 grams for weight > 150 kg. Height is measured to the nearest 1/8 inch using a Schorr stadiometer (Schorr Productions, Olney, MD). Serum creatinine, estimated glomerular filtration rate, hemoglobin A1C, HDL-C, and total cholesterol values are processed by Labcorp, Burlington, NC.

**Table 2:** Heart healthy lenoir patient and practice level measures*

**Variable**	**Instrument**	**Time**	
		**Baseline**	**Follow-up**
Patient level measures			
Blood pressure	Average of 3 readings	X	X^a^
Literacy	Short-TOFHLA	X	
Socio-demographic characteristics	Patient self-report/survey (Self Report)		
Age	Self-report	X	
Gender	Self-report	X	
Marital status	Self-report	X	
Education (grade level)	Self-report	X	
Race/ethnicity (self-report)	Self-report	X	
Household income	Self-report	X	
Employment	Self-report	X	X^f^
Relative social position	MacArthur Scale	X	
Health insurance	Self-report	X	X^c^
Social support	Medical outcomes study/perceived social support	X	
Biometrics			
Height	Stadiometer	X	
Weight	SECA scale	X	X^a^
Creatinine/GFR	Laboratory test	X	X^c^
Cholesterol	Laboratory test	X	X^c^
A1c	Laboratory test	X	X^d^
Clinical characteristics			
Medical comorbidities: Heart failure/high Cholesterol/Lung Disease/Chronic Back Pain/ Cancer/Arthritis/Fibromyalgia/Diabetes/Hypertension/COPD/Obstructive Sleep Apnea/ Renal Insufficiency/Stroke	Self-report	X	
Depression	Mental Health Inventory (MHI-5)	X	X^e^
Smoking status	Self-report	X	X^a^
Current medications and supplements	List generated	X	X^a^
Patient reported outcomes			
Quality of life survey	SF-12	X	X^a^
Medication side-effects that limit use	Self-report	X	X^c^
Knowledge/behaviors/beliefs	Self-report	X	X^a^
Medication adherence (ADH)	Morisky adherence score	X	X^a^
Exercise	RESIDE	X	X^d^
Diet	Block fruit-vegetable-fiber screener	X	X^d^
Understanding illness	HTN beliefs questionnaire	X	X^c^
Participatory decision making (PDM)	PDM survey	X	X^c^
Patient activation	Short- patient activation measure	X	X^a^
Social determinants of health	Social determinants and civic engagement questionnaire		X^b^
Access to medication	Cost-related access to medication survey	X	X^a^
Practice Level QI Process Variables (Selected Visit planner items)			
Action taken if BP uncontrolled	Visit Planner data	Continuous	
Consequences of uncontrolled HTN discussed	Visit Planner data	Continuous	
Assess medication adherence	Visit Planner data	Continuous	
Assess for understanding of instructions	Visit Planner data	Continuous	
Percent patients with HTN with BP’s < 140 mmHg and < 90 mmHg	Performance reports	Monthly and yearly	

^a^Visits with study personnel at 6,12,18 and 24 months post enrollment visit.

^b^only measured at 6 month visit.

^c^measured at 12 and 24 months.

^d^measured at 6, 12, 24 months.

^e^measured at 12, 18, 24 months.

^f^measured at 12 months.

*All patients are given the option to have the study documents read to them.

### Study design: choice of a cohort design

In our early discussions with stakeholders representing the health care workforce in this region, we learned that practice providers and staff members, the majority of whom had never participated in research, were most comfortable with designing the HHL study using a quality improvement approach and one that would have a limited impact on patient flow. Because the study targeted practice level and patient level changes, the risk of contamination of a control group was high. The community also desired to avoid withholding the patient level phone-based coaching component from a control group.

Non-randomized observational trial designs are increasingly used for the evaluation of quality improvement and/or practice-based interventions where rigorous and high fidelity interventions are not feasible or desirable [15,16]. Thus we determined that a cohort design best met the needs of this study.

### Using a community based participatory approach

We chose to use a CBPR approach to inform the design and implementation of this study as it offers a useful method for initial and continued practice engagement in the research process and allows for targeted adaptation of the research activities both within and among practices [17]. During the formative phase of the study, we interviewed multiple stakeholder groups, including staff members, providers, and patients from the participating practices to help design the practice and patient level intervention. We continue to use CBPR in the phasic roll out of the Practice Intervention in order to minimize practice burden, introduce new elements only once practices have demonstrated confidence in taking on new activities, and to ensure that the practice staff members and providers continue to have a voice in designing the ongoing study related activities.

### Qualitative data gathering

#### Formative phase practice interviews

We generated a list of evidenced-based practice interventions that have previously demonstrated a reduction in blood pressure or an increase in control of blood pressure in diverse patient populations. Our domains and questions for the formative phase interview guides drew mainly from Cochrane reviews and the quality improvement literature [18,19]. We provided short summaries of potential intervention activities in order to generate ideas regarding the acceptability and/or feasibility of implementing: 1) team based care, 2) home blood pressure monitoring protocols, 3) processes to address medication adherence, 4) a patient intervention coaching program, and 5) the use of standardized care templates.

We conducted the interviews in two groups at each practice; one with practice leadership (practice management, lead nurses, and providers) and the other with practice staff (front office personnel, billing staff, and other nursing/clinical staff) to maximize the diversity of perspectives and minimize single source bias.

### Formative phase patient stakeholder interviews

We conducted face-to-face interviews with 8–10 hypertensive patients from each practice. Patients for this activity were purposely sampled to include a diversity of race, gender, health literacy and blood pressure control. Locally hired research staff members conducted the interviews. Attempts were made to match interviewer/interviewee by race; this occurred in over 90% of the interviews. The interviews explored domains of overall health concerns, general hypertension knowledge and regional barriers to controlling hypertension. Patients were asked two questions regarding their experiences with health inequity. The detailed results of the practice and patient interviewers are being submitted for publication elsewhere.

#### Conceptual model to enhance blood pressure control

By combining research tested approaches to hypertension management with our qualitative research in the community, we developed a conceptual model of care that we predict will improve hypertension control and reduce disparities in the targeted community. The model works at several levels to change practice and patient behavior. For the practice level intervention, we adapted the Promoting Action on Research Implementation in Health Services (PARIHS) model to conceptualize practice change [13,17]. The practices and the community based health coach are taught communication and behavior change strategies based on Social Cognitive Theory [20], the Transtheoretical Model [21] and Motivational Interviewing [22,23]. The operationalization of these theories into our intervention activities are described below and the overall schema based upon the formative year experience is presented graphically in Figure 2.

Key practice level content areas include developing and enhancing skills in performing the following activities: 1) using a medication algorithm to guide selection of anti-hypertensive medications and other therapeutic recommendations, 2) using team approaches to assist patients with medication adherence, 3) reviewing techniques to accurately measure blood pressure, 4) abstracting and reflecting upon clinical performance data, 5) devising processes to standardize care delivery including decision support, and 6) incorporating health coaching strategies in their care delivery.

Key patient level content areas include partnering with a health coach to enhance knowledge and confidence regarding the use of home blood pressure monitoring, establishing health goals, learning how to participate in their care decisions, and improving medication adherence.

### Practice and patient interventions

#### Practice intervention component 1: the “design team”

We invited a member from each practice to participate in our “design team” that meets via conference call every 1–2 months throughout the formative and implementation phases. During the design team calls, the study team and the practice representatives address general study issues and fine-tune study procedures and tools that are then implemented in the practice settings and discussed with the larger audience of staff members and providers at the regional dinner meetings (see below).

For example, to date the design team has provided suggestions regarding: 1) each of the proposed decision support items included on the visit planner , 2) components of the hypertension medication algorithm, 3) methods for obtaining and sharing the performance data, and 4) content for the regional dinner meeting educational sessions.

#### Practice intervention component 2: quarterly dinner meetings

We use a quarterly dinner meeting format to discuss study related issues and to deliver an educational curriculum regarding QI in hypertension care. All practice staff and providers are invited as well as other stakeholders from the community. The curriculum includes topics such as reviewing performance data noted below, working as teams to standardize care delivery, helping patients better adhere to therapeutic regimens, providing updates on pharmacotherapeutic options for hypertension control, reviewing challenging patient cases, attaining health equity, understanding the principles of health literacy, and understanding and incorporating the role of coaching and motivational interviewing in patient care. Continuing Medical Education credits are made available for participation at the dinner events.

#### Practice intervention component 3: practice facilitation

Each investigator is assigned to a practice and acts as a “facilitator”. The facilitator visits practices every 1–2 months as needed and helps the practices implement study related activities. Practice facilitators engage practice staff and providers in the design and use of decision support and review performance data with them during visits.

#### Practice intervention component 4: measurement of hypertension control and collaborative partnerships

Given that we knew at the outset that the HHL study would coincide with other initiatives such as the Office of the National Coordinator’s (ONC) EHR Incentive program, we partnered with the region’s Area Health Education Center (AHEC) in order to minimize practice burden by aligning our respective data requirements and data standards. We used the same outcome measure of hypertension control as promulgated by the ONC program and endorsed by the national quality forum (NQF 0018; see National Quality Forum - http://www.qualityforum.org). We invite the AHEC stakeholders to all of our dinner meetings and work collaboratively with AHEC partners in offering practice support.

Each practice has a lead IT staff member who is responsible for generating practice level hypertension control data. These members met as a group in the formative phase with one of the HHL investigators to discuss strategies on how to generate this measure from EHR’s and how to examine this data by race and ethnicity. The practice leadership is encouraged to review this data on a monthly basis.

#### Practice intervention component 5: medication algorithm

We created a HHL study hypertension treatment algorithm in the formative phase based on the Seventh Report of the Joint National Committee on Prevention, Detection, Evaluation, and Treatment of High Blood pressure [2] and the AHA/ASH/PCNA Scientific Statement regarding the use of home blood pressure monitors [24]. The investigative team provided the draft algorithm to the Design Team members during two conference calls where edits and recommendations were incorporated. The algorithm accounted for medication costs and specifically included options that were available at the regional pharmacies’ generic “$4” lists. All practice staff and providers reviewed the algorithm at a dinner meeting. The final algorithm document was distributed to the practices and made available on the study’s website (http://www.hearthealthylenoir.com/practices).

#### Practice intervention component 6: design of visit planner/decision support

The use of standardized templates for care delivery was a relatively new concept for the participating practices, thus we planned our roll out for the visit planner implementation over time and in three stages to minimize impact on patient flow. The overall goal is to encourage the practice staff members and providers to function as a team to address blood pressure knowledge, goal setting, and medication adherence with their patients. We also encourage the providers and staff to document the specific actions they take to support the patients’ care in cases where the blood pressure is not well controlled. Providers and clinical staff members are asked to discuss home blood pressure measurements with each patient and the providers are encouraged to take home monitoring data into account when determining whether or not blood pressures are controlled and if actions are required.

Practices are asked to use the visit planners with all of their hypertensive patients one or more days per week. The forms are collected by the research staff monthly and the data is used to document practice changes in practice patterns. We provide this data back to practice leadership and staff members via in office coaching visits and at the quarterly dinner meetings. A copy of our Phase 3 visit planner is available at http://www.hearthealthylenoir.com/practices.

#### Patient intervention component 1: home blood pressure monitoring

Trained research assistants provide instruction on accurate home blood pressure measurement technique based upon methods outlined by The Seventh Report of the Joint National Committee on Prevention, Detection, Evaluation, and Treatment of High Blood Pressure [2] to subjects included in the Patient Intervention. The main instruction occurred at the baseline enrollment visit, but further instruction is provided when deemed appropriate at subsequent study visits or on an ad hoc basis if subjects require additional assistance. Patient Intervention subjects are shown how to document their BP’s in a log book and are instructed to bring the information to each visit with their healthcare provider or research team and to have this information available for phone coaching calls. At a minimum, subjects are asked to record blood pressures three times a week for 2 weeks before each provider visit and to obtain some of these upon wakening and some in the afternoons.

#### Patient Intervention component 2: phone coaching curriculum

We partnered with Bosworth and colleagues to adapt their phone coaching curriculum for the HHL study [25-29]. Slight modifications were made in the original format in order to embed the program content into a linked Microsoft and study tracking database housed at UNC Chapel Hill. Our coach was formally trained in motivational interviewing techniques and completed course work in an Integrative Health Coaching Certification Program. A second coach was trained by the primary coach during year 3 to keep up with call demand. Individual subjects are assigned to one coach.

Each coach makes 4 attempts to reach each subject with the scheduled encounter described in Table 3. The format for the coaching calls includes a review of home blood pressure measurements, medications, and issues that may impact adherence. Each participant and his or her coach review a variety of lifestyle topics and jointly develop realistic goals that are reviewed at subsequent calls. The health coach provides feedback on the participant’s progress and mails targeted resources to the patient when appropriate. The average call time is approximately 15 minutes. A brief one-page summary of each telephone encounter is sent to the subject’s health care provider.

**Table 3:** Phone coaching schedule and content

**Encounter #**	**Module descriptor:**	**Module activities:**
1, 7	Opening _a_	Describe purpose of call, review study
	Medication module _a_, literacy	Review blood pressure medication prescribed, ascertain if taking as prescribed, encourage individuals to contact providers if change in medications, include family member/friends in description of blood pressure medication purpose, information tailored based upon literacy level and specific to blood pressure medications patients are currently taking, suggest questions to ask primary care provider.
	Side effects _a_	Determine side effects experienced and seriousness and discuss
	Memory _b_	Offer mnemonic strategies, explain the importance of taking blood pressure medication consistently
	Closing _a_	Encourage patient to take blood pressure between primary care provider visits
2, 8	Medication module, literacy	Reviews medication changes
	Hypertension knowledge _b_	Educate and address applicable risk factors: diabetes, race, heredity, new diagnosis
	Decision making _b_	Role play to help patient interact with provider more effectively
	Side effects, closing	
3, 9	Diet _c_	Review dietary approaches to stop hypertension* diets. Discuss sodium and food label reading
	Weight _b_	Discuss relationship of weight with hypertension and how to reduce weight
	Medication module, literacy, side effects, closing	
4, 10	Exercise _b_	Provide information based upon stage of change
	Social & medical barriers	Ascertain social support, determine if patient needs referrals, help with refills and provide information about support groups and local resources
	Memory, Medication module, literacy, side effects, closing	
5, 11	Stress _b_	Discuss methods for identifying and reducing stress
	Alcohol _b_	Provide information on the relationship between alcohol use and hypertension. Resources are provided.
	Medication module, literacy, side effects, closing	
6, 12	Medication module, literacy, side effects, memory, closing	
All	Patient initiated	Address patient concern at time of patient initiated call to phone coach.

a = Modules activated at every phone call for only those identified as having a problem.

b = Modules activated at specific intervals for only those identified as having a problem.

c = Modules activated at specific intervals for all individuals.

* Your guide to lowering blood pressure with DASH. National Heart, Lung, and Blood Institute. http://www.nhlbi.nih.gov/health/public/heart/hbp/dash/new_dash.pdf. Accessed April 30, 2013.

### Evaluation - outcome assessment

#### Practice intervention - blood pressure control

We collect practice level data reflecting the percentage of hypertensive patients seen each month who are controlled, defined as having a systolic blood pressure less than 140 mmHg and a diastolic blood pressure less than 90 mmHg at their office visit. Every six months we separately pull data to reflect a full year of BP’s using the most recent recorded BP’s to generate the control rates. The monthly and yearly control data are stratified by race and ethnicity. This data is shared with the stakeholders at our office visits, design team meetings and dinner meetings.

#### Patient intervention – change in blood pressure

Our primary patient level analysis will be based upon changes in blood pressure among individuals in the Patient Intervention. We will evaluate the differential effect of the intervention by race (African American and White) and health literacy level (low versus higher health literacy) on blood pressure.

#### Other patient intervention outcomes measures

In addition to the biometric measures, we assess several social, demographic, and patient reported outcome variables, as outlined in Table 2, that can be analyzed as secondary outcomes.

### Sample size and power calculation: patient intervention

Our primary analyses will be to evaluate the effect of the Patient Intervention on reducing blood pressure and to compare the change in mean systolic blood pressure among African Americans and Whites to test our primary hypothesis that African American patients will have greater improvement in mean systolic blood pressure at the end of 1 year of engaging in the intervention. Based on a previous study [12], we estimated that the within-practice intraclass correlation for change in systolic BP was zero and standard deviation was 16 mmHg. We assumed that the baseline difference in systolic blood pressure between African Americans and Whites would be approximately 5 mmHg. We expect the patient intervention to be effective in reducing systolic blood pressure in both groups, but produce a greater reduction in African Americans by approximately 3.5 mmHg. Table 4 illustrates sample sizes required to detect various differences in mean systolic blood pressure reductions between the two races using one-sided tests of significance at alpha =0.05. With data from 520 subjects, we will have 80% power to detect a difference of 3.5 mmHg in systolic blood pressure reduction between the two races (see in Table 4). Since we expect an attrition rate of approximately 15%, we planned to enroll 600 hypertensive patients (approximately 300 African Americans and 300 Whites). We will have similar power to detect differences in mean systolic blood pressure change between high and low literacy groups, assuming an equal distribution of literacy.

**Table 4:** Sample size estimates to detect various differences in mean changes of systolic BP between African Americans and Whites using a one-sided t-tests with alpha = 0.05 and standard deviation of change =16 mmHg

**Power (1-Beta)**	**Sample size - African Americans**	**Sample size - Whites**	**Difference in mean changes of SBP between two races**
0.80027	792	792	2.0
0.80033	507	507	2.5
0.80023	352	352	3.0
0.80125	260	260	3.5
0.80086	199	199	4.0
0.80002	157	157	4.5
0.80193	128	128	5.0

## Discussion

We designed the HHL study using an emerging methodology that combines the principles of practice based research and community based participatory research, the combination of which is felt to hold great promise for moving the bar towards achieving health equity [30] and enhancing awareness regarding issues faced by that marginalized communities [10]. The study design will allow us to evaluate the effect of combined practice level and individual level interventions on blood pressure control and to assess for differential impact of such a program by race and literacy levels. CBPR informed interventions have been successful in reducing other cardiovascular risk factors [3,31] and are increasingly focusing on hypertension as a main outcome [4,32]. Notably, in one such CBPR trial that used an analogous multi-level intervention, trends were noted in improved blood pressure among the subjects with uncontrolled hypertension [32]. The findings of our study will complement this important work, and contribute to our understanding of the effectives of CBPR-based interventions in lowering blood pressure.

Our process has been to adapt evidence-based approaches to the local context using the resources available. We have already seen immediate impact and dissemination through our relationship with the region’s Medicaid Network organization. They have adapted the phone coaching curriculum to support a new program that helps patients create personal health plans, a key self-management resource for patients with hypertension, but also a key component of the practices’ efforts to become patient centered medical homes. By building numerous partnerships, we are confident that many significant resources will remain in this community long after the HHL study ends. These relationships have also built the foundation for further health services innovations in this region.

Although we are still in the data collection and implementation phase of the study, we can share several observations of this work to date. First, the local community is genuinely interested in the research process and has contributed to the development of the interventions. The practices helped us recruit patients for and also participated in qualitative interviews to allow the research team to better understand the needs and resources of the primary care environment. They continue to give guidance in the design team calls and this feedback drives the evolution of the Practice Intervention and the process of linking the work of the community coach to the practice team.

Second, we have had variable levels of engagement in the practices to date. When we initially solicited the participation of local primary care providers, only half of the practices agreed to join the project. Among the 6 practices currently in the project, engagement is high among 2, moderate among 2, and low among 2. We are still in the implementation phase and are taking steps to increase engagement and to be responsive to the unique challenges faced by each individual practice. Enhancing primary care in a resource-limited rural community presents challenges with setting up an innovative network of practices that are not geographically close and establishing ties that facilitate rapid dissemination and implementation of the interventions. Third, there has been a high turnover rate in some practices. In two practices, none of the original physicians, physician assistant and nurse practitioners remain. This level of turnover presents important limitations to diffusion of information within the practice, particularly in this setting where provider leadership is critical to success.

Fourth, although all of the practices in this community have EHR’s, using the EHR to drive improvements in care delivery and measurement of care outcomes has not been straightforward. None of the practices were able to pull accurate population health data from their EHR at the onset of the study. With substantial effort from our study team and the AHEC’s Regional Extension Center, 4 out of 6 practices can now produce this data. The challenge of adequate implementation and use of EHRs to realize any specific benefits to care deliver should not be underestimated.

We look forward to analysis of study data and exploration of findings with the community. We anticipate end of data collection in late 2014 and analyses in early 2015.

## Abbreviations

AHEC: Area health education center; CBPR: Community based participatory research; CVD: Cardiovascular disease; EHR: Electronic health record; HDL-C: High density lipoprotein-cholesterol; HHL: Heart healthy lenoir; LHL: Low health literacy; NC: North Carolina; ONC: Office of the national coordinator; PARIHS: Promoting action on research implementation in health services; QI: Quality improvement; s-TOFLA: Short test of functional health literacy in adults.

## Competing interests

The authors declare that they have no financial or non-financial competing interests.

## Authors’ contributions

All authors on the paper have made substantial contributions to the manuscript, including matters related to conceptualization, discussing and drafting subsections of the manuscript. All authors reviewed and approved the final version.

## Pre-publication history

The pre-publication history for this paper can be accessed here:

http://www.biomedcentral.com/1472-6963/13/441/prepub

## References

[B1] CDC Vital signs: awareness and treatment of uncontrolled hypertension among adults--United States, 2003–2010 MMWR Morbidity and mortality weekly report 2012 13 703 709 22951452

[B2] Chobanian AV Bakris GL Black HR Cushman WC Green LA Izzo JL Jr Jones DW Materson BJ Oparil S Wright JT Jr The seventh report of the joint national committee on prevention, detection, evaluation, and treatment of high blood pressure: the JNC 7 report Jama 2003 13 19 2560 2572 12748199 10.1001/jama.289.19.2560

[B3] Wilcox S Laken M Bopp M Gethers O Huang P McClorin L Parrott AW Swinton R Yancey A Increasing physical activity among church members: community-based participatory research Am J Prev Med 2007 13 2 131 138 17234487 10.1016/j.amepre.2006.10.009

[B4] Wilcox S Laken M Parrott AW Condrasky M Saunders R Addy CL Evans R Baruth M Samuel M The faith, activity, and nutrition (FAN) program: design of a participatory research intervention to increase physical activity and improve dietary habits in African American churches Contemporary clinical trials 2010 13 4 323 335 20359549 10.1016/j.cct.2010.03.011 PMC2899891

[B5] Burt VL Whelton P Roccella EJ Brown C Cutler JA Higgins M Horan MJ Labarthe D Prevalence of hypertension in the US adult population. Results from the Third National Health and Nutrition Examination Survey, 1988–1991 Hypertension 1995 13 3 305 313 7875754 10.1161/01.hyp.25.3.305

[B6] Hall WD Ferrario CM Moore MA Hall JE Flack JM Cooper W Simmons JD Egan BM Lackland DT Perry M Jr Hypertension-related morbidity and mortality in the southeastern United States Am J Prev Med 1997 13 4 195 209 10.1097/00000441-199704000-00002 9099149

[B7] Klag MJ Whelton PK Randall BL Neaton JD Brancati FL Stamler J End-stage renal disease in African-American and white men. 16-year MRFIT findings Jama 1997 13 16 1293 1298 9109467

[B8] Singh GKK MacDorman M Advance report of final mortality statistics Mon Vital Stat Rep 1994 13 1 76

[B9] El Assaad MA Topouchian JA Darne BM Asmar RG Validation of the Omron HEM-907 device for blood pressure measurement Blood Pres Monit 2002 13 4 237 241 10.1097/00126097-200208000-00006 12198340

[B10] Savage CL Xu Y Lee R Rose BL Kappesser M Anthony JS A case study in the use of community-based participatory research in public health nursing Public Health Nurs 2006 13 5 472 478 16961566 10.1111/j.1525-1446.2006.00585.x

[B11] White WB Anwar YA Evaluation of the overall efficacy of the Omron office digital blood pressure HEM-907 monitor in adults Blood Pres Monit 2001 13 2 107 110 10.1097/00126097-200104000-00007 11433132

[B12] Sheridan SL Draeger LB Pignone MP Keyserling TC Simpson RJ Jr Rimer B Bangdiwala SI Cai J Gizlice Z A randomized trial of an intervention to improve use and adherence to effective coronary heart disease prevention strategies BMC Health Serv Res 2011 13 331 22141447 10.1186/1472-6963-11-331 PMC3268742

[B13] Kitson AL Rycroft-Malone J Harvey G McCormack B Seers K Titchen A Evaluating the successful implementation of evidence into practice using the PARiHS framework: theoretical and practical challenges Implement Sci 2008 13 1 18179688 10.1186/1748-5908-3-1 PMC2235887

[B14] Davis TC Wolf MS Bass PF 3rd Thompson JA Tilson HH Neuberger M Parker RM Literacy and misunderstanding prescription drug labels Ann Intern Med 2006 13 12 887 894 17135578 10.7326/0003-4819-145-12-200612190-00144

[B15] Handley MA Schillinger D Shiboski S Quasi-experimental designs in practice-based research settings: design and implementation considerations J Am Board Fam Med 2011 13 5 589 596 21900443 10.3122/jabfm.2011.05.110067

[B16] Bonell CP Hargreaves J Cousens S Ross D Hayes R Petticrew M Kirkwood BR Alternatives to randomisation in the evaluation of public health interventions: design challenges and solutions J Epidemiol Community Health 2011 13 7 582 587 19213758 10.1136/jech.2008.082602

[B17] Rycroft-Malone J Kitson A Harvey G McCormack B Seers K Titchen A Estabrooks C Ingredients for change: revisiting a conceptual framework Qual Saf Health Care 2002 13 2 174 180 12448812 10.1136/qhc.11.2.174 PMC1743587

[B18] Walsh J McDonald KM Shojania KG Sundaram V Nayak S Davies S Lewis R Mechanic J Sharp C Henne M Closing the quality gap: A critical analysis of quality improvement strategies. Technical Review 9. (Prepared by the Stanford University-UCSF Evidence-Based Practice Center, under Contract No. 290-02-0017. AHRQ Publication No 04-0051-3 2005 Rockville, MD: Agency for Healthcare Research and Quality 20734527

[B19] Glynn LG Murphy AW SMS Schroeder K Fahey T Interventions used to improve control of blood pressure in patients with hypertension Cochrane Database Syst Rev 2010 Issue 3 Art. No.:CD005182.DOI10.1002/14651858.CD005182.pub4 10.1002/14651858.CD005182.pub4 PMC13248079 20238338

[B20] Bandura Social Foundations of Thought and Action: A Social Cognitive Theory 1986 Englewood Cliffs, NJ: Prentice Hall

[B21] Prochaska JO DiClemente CC Stages and processes of self-change of smoking: toward an integrative model of change J Consult Clin Psychol 1983 13 3 390 395 6863699 10.1037//0022-006x.51.3.390

[B22] Rollnick SMW What is Motivational Interviewing? Behav Cogn Psychother 1995 13 4 325 334

[B23] Miller WR Rollnick S Motivational Interviewing: Preparing People for Change 2002 2 New York, NY: The Guilford Press

[B24] Pickering TG Miller NH Ogedegbe G Krakoff LR Artinian NT Goff D Call to action on use and reimbursement for home blood pressure monitoring: executive summary: a joint scientific statement from the American Heart Association, American Society Of Hypertension, and Preventive Cardiovascular Nurses Association Hypertension 2008 13 1 1 9 18497371 10.1161/HYPERTENSIONAHA.107.189011

[B25] Bosworth HB Olsen MK Dudley T Orr M Neary A Harrelson M Adams M Svetkey LP Dolor RJ Oddone EZ The Take Control of Your Blood pressure (TCYB) study: study design and methodology Contemporary clinical trials 2007 13 1 33 47 16996808 10.1016/j.cct.2006.08.006

[B26] Bosworth HB Olsen MK Grubber JM Neary AM Orr MM Powers BJ Adams MB Svetkey LP Reed SD Li Y Two self-management interventions to improve hypertension control: a randomized trial Ann Intern Med 2009 13 10 687 695 19920269 10.1059/0003-4819-151-10-200911170-00148 PMC2892337

[B27] Bosworth HB Olsen MK McCant F Harrelson M Gentry P Rose C Goldstein MK Hoffman BB Powers B Oddone EZ Hypertension Intervention Nurse Telemedicine Study (HINTS): testing a multifactorial tailored behavioral/educational and a medication management intervention for blood pressure control American heart journal 2007 13 6 918 924 17540191 10.1016/j.ahj.2007.03.004

[B28] Bosworth HB Oddone EZ A model of psychosocial and cultural antecedents of blood pressure control J Natl Med Assoc 2002 13 4 236 248 11991336 PMC2594228

[B29] Bosworth HB Olsen MK Goldstein MK Orr M Dudley T McCant F Gentry P Oddone EZ The veterans’ study to improve the control of hypertension (V-STITCH): design and methodology Contemporary clinical trials 2005 13 2 155 168 15837438 10.1016/j.cct.2004.12.006

[B30] Eriksen M Rothenberg R Community-based participatory research (CBPR). Editorial Health Educ Res 2012 13 4 553 554 22798615 10.1093/her/cys066

[B31] Kim KH Linnan L Campbell MK Brooks C Koenig HG Wiesen C The WORD (wholeness, oneness, righteousness, deliverance): a faith-based weight-loss program utilizing a community-based participatory research approach Health Educ Behav 2008 13 5 634 650 17200103 10.1177/1090198106291985

[B32] Cooper LA Roter DL Carson KA Bone LR Larson SM Miller ER 3rd Barr MS Levine DM A randomized trial to improve patient-centered care and hypertension control in underserved primary care patients J Gen Intern Med 2011 13 11 1297 1304 21732195 10.1007/s11606-011-1794-6 PMC3208476

